# Effects of CYP3A4*22 and POR*28 variations on the pharmacokinetics of tacrolimus in renal transplant recipients: a meta-analysis of 18 observational studies

**DOI:** 10.1186/s12882-024-03467-4

**Published:** 2024-02-06

**Authors:** Ze Li, Xiaozhen Wang, Dandan Li, Sheng Cheng, Zhe Li, Heng Guo, Yiwen Dong, Yingming Zheng, Xingang Li

**Affiliations:** 1 Department of Pharmacy, Beijing Friendship Hospital, Capital Medical University, 95 Yong An Road, Xi Cheng District, Beijing, China; 2 Central Laboratory, Xuanwu Hospital, Capital Medical University, Beijing, China

**Keywords:** Meta-analysis, Genetic polymorphisms, Tacrolimus, Pharmacokinetics, Renal transplant recipients

## Abstract

**Purpose:**

This study aimed to investigate the association between cytochrome P450 (CYP) 3A4*22 and cytochrome P450 oxidoreductase (POR)*28 variations and the pharmacokinetics of tacrolimus.

**Methods:**

Cochrane Central Register of Controlled Trials (CENTRAL), Web of Science (SCI), MEDLINE, and Embase were systematically searched from inception to August 2022. The outcomes were weight-adjusted daily dose and dose-adjusted trough concentration (C_0_/Dose).

**Results:**

The study included 2931 renal transplant recipients from 18 publications. Weight-adjusted daily dose of CYP3A4*1/*1 carriers was 0.04 (WMD = 0.04, 95% CI: 0.02 to 0.06), 0.03 (WMD = 0.03, 95% CI: 0.02 to 0.05), 0.02 (WMD = 0.02, 95% CI: 0.01 to 0.03), or 0.02 mg/kg/day (WMD = 0.02, 95% CI: 0.00 to 0.04) higher than CYP3A4*22 carriers in Caucasians at 1 month, 3 months, 6 months, or 12 months post-transplantation. Conversely, C0/Dose was lower for CYP3A4*1/*1 carriers at 3 days (SMD = -0.35, 95% CI: -0.65 to -0.06), 1 month (SMD = -0.67, 95% CI: -1.16 to -0.18), 3 months (SMD = -0.60, 95% CI: -0.89 to -0.31), 6 months (SMD = -0.76, 95% CI: -1.49 to -0.04), or 12 months post-transplantation (SMD = -0.69, 95% CI: -1.37 to 0.00). Furthermore, C_0_/Dose of POR*1/*1 carriers was 22.64 (WMD = 22.64, 95% CI: 2.54 to 42.74) or 19.41 (ng/ml)/(mg/kg/day) (WMD = 19.41, 95% CI: 9.58 to 29.24) higher than POR*28 carriers in CYP3A5 expressers at 3 days or 7 days post-transplantation, and higher in Asians at 6 months post-transplantation (SMD = 0.96, 95% CI: 0.50 to 1.43).

**Conclusions:**

CYP3A4*22 variant in Caucasians restrains the metabolism of tacrolimus, while POR*28 variant in CYP3A5 expressers enhances the metabolism of tacrolimus for renal transplant recipients. However, further well-designed prospective studies are necessary to substantiate these conclusions given some limitations.

**Supplementary Information:**

The online version contains supplementary material available at 10.1186/s12882-024-03467-4.

## Introduction

End-stage renal disease (ESRD) affected an increasing number of patients worldwide, most of whom relied on dialysis therapy [1]. However, kidney transplantation remained the optimal treatment option, offering improved quality of life and reduced costs [2]. Immunosuppressant drugs were used in transplantation therapy to prevent immune system attacks on newly transplanted organs [3]. One such drug, tacrolimus, as an immunosuppressant cornerstone, discovered in 1984 and utilized in 1989 [4], had been widely used in organ transplantation treatment possessing a wide range of intra- and inter-individual pharmacokinetics variability and narrow therapeutic window. Consequently, therapeutic drug monitoring (TDM) was routinely conducted to maintain the target range and avoid overexposure, which caused toxicity including nephrotoxicity, hypertension, or neurotoxicity [5]. However, TDM was not convenient or powerful in determining the appropriate initial dose. At present, Several clinical pharmacokinetic factors influencing tacrolimus have been identified, including food consumption, diarrhea, hemolytic anemia, hepatic and kidney disorders, and genetic polymorphisms [6].

Genetic polymorphisms, significantly affecting tacrolimus dose requirements and systemic exposure in renal transplant recipients, played an important role in predicting the initial tacrolimus dosage [7]. A few meta-analyses had shown an association between genetic polymorphisms of cytochrome P450 (CYP) or the ATP Binding Cassette Subfamily B Member 1 (ABCB1) and the pharmacokinetics of tacrolimus in the renal transplant recipients. For example, tacrolimus trough blood concentration/Dose (C_0_/Dose) ratio was significantly lower in CYP3A5*1, CYP3A4*1B or *1G, or ABCB1 3435CC carriers than CYP3A5*3/*3, CYP3A4*1/*1, or ABCB1 3435 T carriers, and CYP3A4*1/*1 or ABCB1 3435TT carriers required a lower weight-adjusted tacrolimus daily dose compared to CYP3A4*1B or *1G, or ABCB1 3435CC carriers [8–12]. However, the selection of the optimal initial dose and dose adjustment based on the genetic background were still not widely performed in clinical practice due to insufficient clinical evidence [13].

Up to now, researchers were attracted by some genetic polymorphisms such as CYP3A4*22 (poor metabolizer) or cytochrome P450 oxidoreductase (POR) *28 (extensive metabolizer) variants, but the impact on the pharmacokinetics of tacrolimus remained controversial. Actually, a meta-analysis demonstrated a definite correlation between the POR*28 genotype and the pharmacokinetics of tacrolimus, emphasizing the POR*28 carriers required a higher dose of tacrolimus to achieve target levels compared to those with POR*1/*1 [14]. However, as the author stated, the meta-analysis existed some limitations, firstly, it only focused on the impact of POR polymorphism in the early stage of transplantation, with six studies, lacking investigation on other processes of the transplantation; secondly, due to the small number of included studies, subgroup analysis stratified by the ethnicity cannot be conducted. Therefore, the aim of our study is to conduct a comprehensive meta-analysis by searching and screening eligible studies involving these genetic variations, providing an insight into tacrolimus dose adjustment based on preemptive genotyping results, and we hope that our research will address the limitations of the previous meta-analysis and provide more robust and reliable results.

## Methods

### Search strategy and study selection

A comprehensive search was performed in the Cochrane Central Register of Controlled Trials (CENTRAL), Web of Science (SCI), MEDLINE, and Embase databases from inception to August 2022, using a developed search strategy that was specifically designed for this meta-analysis. Details of the search strategy are provided in Supplementary Table S1.

Screening criteria were developed for this meta-analysis prospectively. The inclusion criteria were as follow: (1) the target population consisted of adult renal transplant recipients; (2) studies involved the administration of tacrolimus as a treatment; (3) studies reported outcomes that included weight-adjusted daily dose or C_0_/Dose; (4) studies included genotyping results. Exclusion criteria were applied to patients with combined organ transplantations, exposure to cyclosporine or intravenous tacrolimus, co-administration of azole antifungal agents, or who were under 18 years old. We also conducted a manual search of the reference lists of included studies and relevant meta-analyses to identify additional eligible studies.

### Data extraction and quality assessment

This meta-analysis was conducted in adherence to the PRISMA (Preferred Reporting Items for Systemic Reviews and Meta-analysis) and MOOSE (Meta-analysis of Observational Studies in Epidemiology) guidelines [15, 16].

The outcomes were weight-adjusted daily dose and C_0_/Dose. Two independent reviewers (Ze Li and Xiaozhen Wang) were responsible for screening the titles and abstracts of retrieved studies to ensure they met the criteria for inclusion. Full-text articles of the remaining studies were then screened by the same reviewers to identify those that met all of the inclusion criteria. We also conducted a manual search of the reference lists of each article to identify relevant studies. For each included study, two reviewers independently extracted the characteristics and outcomes using a predefined data table. Any discrepancies were resolved through consultation with the supervisor (Xingang Li).

Bias risks were assessed with the quality checklist derived from the Strengthening the Reporting of Genetic Association (STREGA) recommendations for reports on genetic association studies [17]. The publication bias was quantitatively assessed by the Egger’s test [18], and *P* < 0.05 was taken as statistically significant. Two reviewers (Ze Li and Xiaozhen Wang) assessed risks of bias independently and in duplicate, and any disagreements were resolved through consultation with the supervisor (Xingang Li).

### Data synthesis and statistical analysis

All analyses were performed by Stata 16.0 (StataCorp, College Station, TX, 77,845, USA). The continuous data with the same measurement unit were pooled by weighted mean difference (WMD) and 95% confidence interval (CI) with the random-effect model. If the measurement units were different, the data were pooled by the standard mean difference (SMD). In cases where the data were presented as a median and range or quantile, we applied a special mathematical method to convert the data into the mean and standard deviation to perform meta-analysis indirectly [19]. The heterogeneity among studies was assessed by *I*^*2*^, with < 25%, 25 ~ 50%, and > 50% indicating low, moderate, and high degrees of heterogeneity, respectively. To explore the discrimination in outcomes based on various factors, such as time courses of post-transplantation, combined genotype, or ethnicity, subgroup meta-analyses were performed by stratifying patients into specific relevant groups. For all comparisons in this meta-analysis, statistical significance was defined as *P* < 0.05.

## Results

### Identification and characteristics of studies

**Fig. 1:**
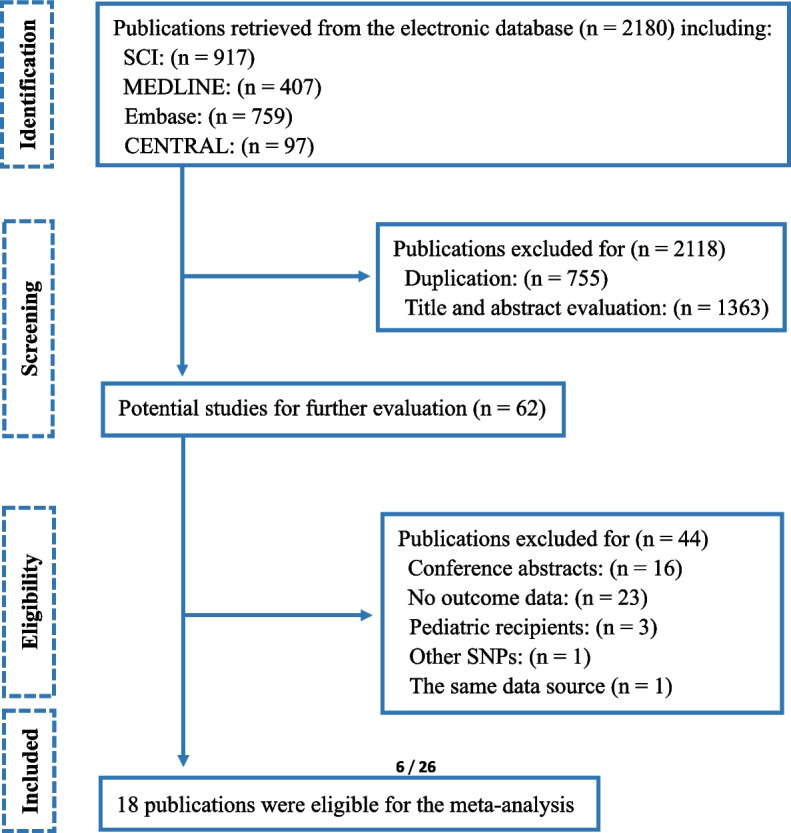
Flow-chart of the studies screening. SNP: single nucleotide polymorphism

A total of 2180 publications were identified through the database search (Fig. 1), and eighteen studies meeting the inclusion criteria were enrolled after the screening process.

Summarily, the meta-analysis included a total of 2,931 renal transplant recipients across 18 studies. Specifically, 1,489 patients were enrolled in the CYP3A4*22 group, and 1,862 patients were enrolled in the POR*28 group. Details of the baseline characteristics of the included studies are presented in Table 1.

**Table 1:** Patient baseline characteristics of included studies

**Author, year**	**Ethnicity**	**Genotype**	**Sample size**	**Age (year)**	**Male (%)**	**Weight (Kg)**	**Genotype method**	**Measurement unit**	**Tacrolimus measurement method**	**Time course for tacrolimus measurement**	**Initial dosage of tacrolimus**	**Target trough level, ng/mL**	**immunosuppressive drugs therapy**
Kuypers D, 2014 [20]	Caucasian	CYP3A4*22, POR*28	22	52.8 ± 13.3	60.0	70.6 ± 14.3	TaqMan assay	mg/kg/day, ng/ml/mg	MEIA	3, 7, 30, 90, 360 d	0.2 mg/kg/day	10 ~ 15	MMF, methylprednisolone
Cheng F, 2021^a^ [21]	Asian	POR*28	201	42.0 ± 11.9	73.3	21.3 ± 2.4^b^	CBT-PMRA Kit	ng/ml/mg	MEIA	30, 90, 360 d	3.0 ~ 5.0 mg, bid	8 ~ 10	MMF
Bruckmueller H, 2015 [22]	Caucasian	CYP3A4*22, POR*28	223	48.0 ± 14.0	58.7	NR	TaqMan assay	ng/ml/mg	LC–MS	NR	NR	NR	NR
Tavira B, 2013^a^ [23]	Caucasian	CYP3A4*22	206	48.6 ± 13.2	NR	25.3 ± 4.9^b^	TaqMan assay	mg/kg/day, (ng/ml)/ (mg/kg/day)	MEIA	7, 180 d	0.2 mg/kg/day	10 ~ 15	MMF, prednisone
Madsen MJ, 2017^a^ [24]	Caucasian	CYP3A4*22, POR*28	52	49.2 ± 12.3	57.7	77.0 ± 20.0	TaqMan assay	ng/ml/mg	MEIA	180, 360 d	NR	NR	NR
Lunde I, 2014^a^ [25]	Caucasian	CYP3A4*22, POR*28	123	48.8 ± 9.8	70.0	87.6 ± 19.1	PCR–RFLP	ng/ml/mg	MEIA	NR	NR	3 ~ 7	MMF, steroids
Liu S, 2016 [26]	Asian	POR*28	154	40.0 ± 10.9	NR	59.8 ± 10.7	Agena Bioscience MassARRAY system	(ng/ml)/ (mg/kg/day)	MEIA	7 d	0.05 ~ 0.075 mg/kg, bid	5 ~ 8	MMF, prednisone
Kurzawski M, 2014^a^ [27]	Caucasian	POR*28	241	45.8 ± 12.4	44.4	73.2 ± 13.9	TaqMan assay	(ng/ml)/ (mg/kg/day)	MEIA	7, 30, 90, 180, 360 d	0.1 mg/kg/day	10 ~ 15	MMF, steroids
Kurzawski M, 2014 [28]	Caucasian	CYP3A4*22	241	45.8 ± 12.4	44.4	73.2 ± 13.9	TaqMan assay	mg/kg/day, (ng/ml)/ (mg/kg/day)	MEIA	3, 30, 90, 180, 360 d	0.1 mg/kg/day	10 ~ 15	MMF, steroids
Elens L, 2011 [29]	Caucasian, Asian	CYP3A4*22	185	47.9 ± 13.8	61.0	72.6 ± 14.0	TaqMan assay	mg/kg/day, (ng/ml)/ (mg/kg/day)	MEIA	3, 30, 90, 180, 360 d	NR	5 ~ 12	corticosteroids
Vanhove T, 2017 [30]	Caucasian	CYP3A4*22	279	53.0 ± 13.0	60.0	73.4 ± 15.2	OpenArray platform	ng/ml/mg	LC–MS	3, 7, 14 d	0.2 mg/kg/day	12 ~ 15	MMF, methylprednisolone
Jonge H, 2014 [31]	Caucasian	CYP3A4*22	59	54.2 ± 10.9	60.0	71.4 ± 13.7	TaqMan assay	mg/kg/day, ng/ml/mg	LC–MS	NR	0.2 mg/kg/day	10 ~ 15	MMF, methylprednisolone
Elens L, 2011 [32]	Caucasian	CYP3A4*22	99	50.5 ± 13.2	62.0	73.6 ± 13.2	TaqMan assay	mg/kg/day, (ng/ml)/ (mg/kg/day)	MEIA	NR	NR	5 ~ 15	MMF, prednisone, methylprednisolone
Zhang JJ, 2015 [33]	Asian	POR*28	83	40.4 ± 11.3	72.3	62.0 ± 9.4	PCR–RFLP	(ng/ml)/ (mg/kg/day)	MEIA	3, 7 d, NR	NR	10 ~ 15	MMF, methylprednisolone
Li CJ, 2014 [34]	Asian	POR*28	240	41.0 ± 12.2	67.1	57.9 ± 10.1	Applied Biosystems Multiplex Kit	(ng/ml)/ (mg/kg/day)	MEIA	3, 7, 14 d	0.1 mg/kg, bid	10 ~ 12	MMF, steroids
Phupradit A, 2018 [35]	Asian	POR*28	216	43.0 ± 14.6	61.1	57.1 ± 11.3	TaqMan assay	(ng/ml)/ (mg/kg/day)	MEIA	7 d	0.05 mg/kg, bid	4 ~ 8	MMF, corticosteroids
Elens L, 2014^a^ [36]	Caucasian	POR*28	184	49.5 ± 15.3	60.2	72.6 ± 15.7	TaqMan assay	(ng/ml)/ (mg/kg/day)	MEIA	3, 30, 90, 180, 360 d	NR	5 ~ 15	MMF, prednisone, methylprednisolone
Si SH, 2018 [37]	Asian	POR*28	123	36.9 ± 9.5	72.0	60.5 ± 9.3	Qubit dsDNA HS Assay Kit	(ng/ml)/(mg/ kg/m^2^ )	Architect I2000 optical detection system	7, 14, 30, 90, 180, 360 d	0.2 mg/kg/day	NR	MMF, sirolimus or prednisone

*MEIA* microparticle enzyme immunoassay, *d* day, *MMF* mycophenolate mofetil, *LC–MS* liquid chromatography–mass spectrometry, *PCR–RFLP* cleaved amplification polymorphism sequence-tagged sites, *NR* not reported

^a^means the value is calculated by the median and range

^b^means the value is shown as BMI (body mass index)

### Risks of bias assessment

The risk of bias across the included studies was evaluated and the results are presented in Supplementary Table S2. Most of the studies displayed low risks of bias. Notably, nine studies [22, 24, 25, 29, 31–33, 36, 37] were found to have incomplete descriptions of the study information, such as a lack of information on time courses of post-transplantation, initial dosage, or target trough level. In addition, four studies [22, 24, 27, 28] did not provide sufficient details regarding eligibility criteria, while five studies [23, 25, 33–35] did not make any reference to the exclusion criteria. Three studies did not report the results of H-W equilibrium [25, 30, 33], and the other three studies [22, 23, 26] involved insufficient demographic data without weight or percent of male. Furthermore, no publication bias was observed by the Egger’s tests except the subgroup analysis of POR*1/*1 versus POR*28 carriers in Caucasian recipients at 6 months post-transplantations (*P* = 0.037). Detailed results of the publication bias assessment are shown in Supplementary Table S3.

### Effects of genetic polymorphisms on weight-adjusted daily dose and C_0_/Dose of tacrolimus

#### CYP3A4*22

**Fig. 2:**
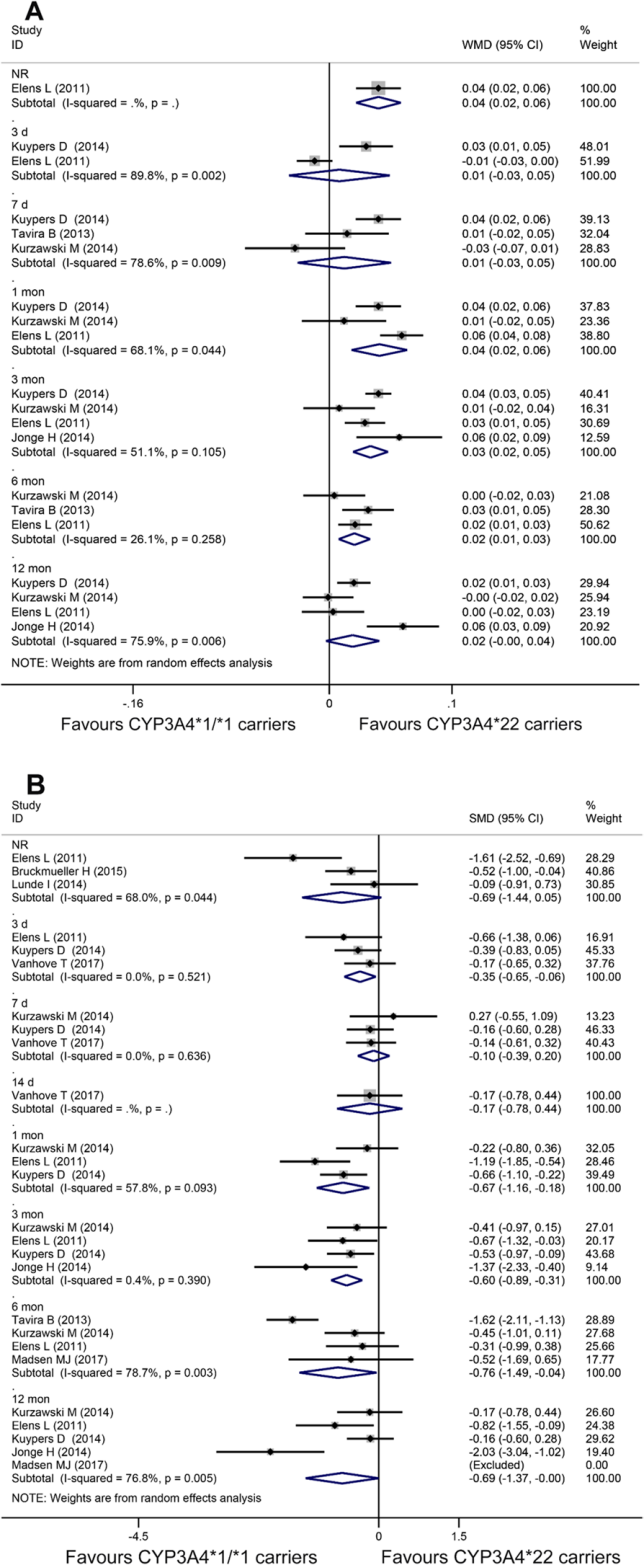
Forest plots illustrating the relationship between CYP3A4 genetic variants and tacrolimus pharmacokinetics. **A** Weight-adjusted daily dose of CYP3A4*1/*1 carriers versus CYP3A4*22 carriers at different post-transplantation time courses; **B** C_0_/Dose of CYP3A4*1/*1 carriers versus CYP3A4*22 carriers at different post-transplantation time courses. C_0_/Dose: dose-adjusted trough concentration; NR: not reported; WMD: weighted mean difference; SMD: standard mean difference; CI: confidence interval; d: days; mon: months

In comparison to CYP3A4*22 carriers, recipients with CYP3A4*1/*1 displayed a weight-adjusted daily dose that was 0.04 (WMD = 0.04, 95% CI: 0.02 to 0.06, *I*^*2*^ = 68.1%), 0.03 (WMD = 0.03, 95% CI: 0.02 to 0.05, *I*^*2*^ = 51.1%), 0.02 (WMD = 0.02, 95% CI: 0.01 to 0.03, *I*^*2*^ = 26.1%), or 0.02 mg/kg/day (WMD = 0.02, 95% CI: 0.00 to 0.04, *I*^*2*^ = 75.9%) higher, respectively, for recipients at 1 month, 3 months, 6 months, or 12 months post-transplantation (Fig. 2A). Similarly, for recipients at 3 days (SMD = -0.35, 95% CI: -0.65 to -0.06, *I*^*2*^ = 0.0%), 1 month (SMD = -0.67, 95% CI: -1.16 to -0.18, *I*^*2*^ = 57.8%), 3 months (SMD = -0.60, 95% CI: -0.89 to -0.31, *I*^*2*^ = 0.4%), 6 months (SMD = -0.76, 95% CI: -1.49 to -0.04, *I*^*2*^ = 78.7%), or 12 months (SMD = -0.69, 95% CI: -1.37 to 0.00, *I*^*2*^ = 76.8%) post-transplantation, a significantly lower C_0_/Dose was observed in CYP3A4*1/*1 carriers compared to CYP3A4*22 carriers (Fig. 2B). However, no significant difference was observed for recipients at the other time courses of post-transplantations in the two comparisons.

#### POR*28

**Fig. 3:**
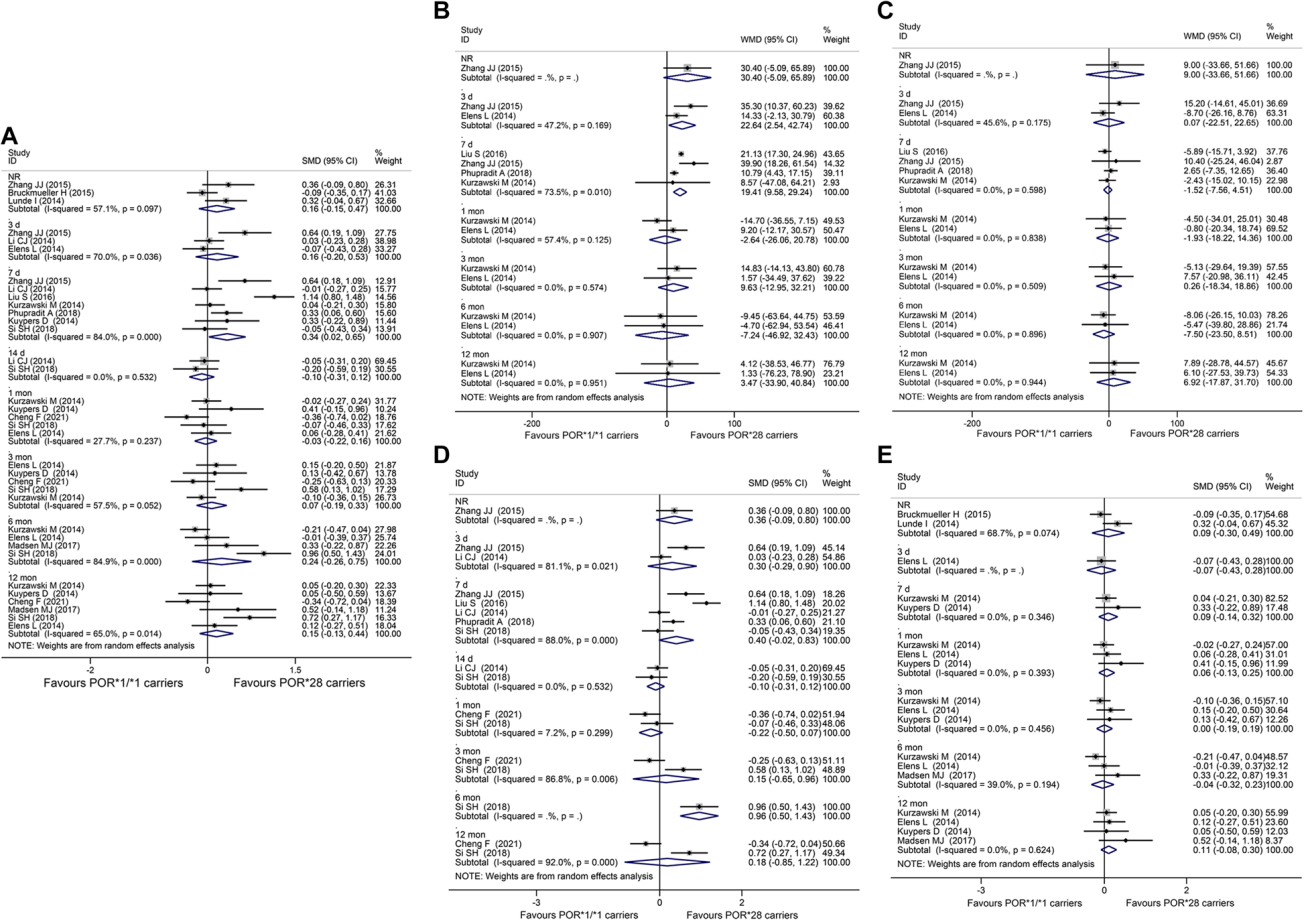
Forest plots illustrating the relationship between POR genetic variants and tacrolimus pharmacokinetics. **A** C_0_/Dose of POR*1/*1 carriers versus POR*28 carriers at different post-transplantation time courses; **B** C_0_/Dose of POR*1/*1 carriers versus POR*28 carriers in CYP3A5 expressers at different post-transplantations time courses; **C** C_0_/Dose of POR*1/*1 carriers versus POR*28 carriers in CYP3A5 non-expressers at different post-transplantations time courses; **D** C_0_/Dose of POR*1/*1 carriers versus POR*28 carriers in Asian recipients at different post-transplantations time courses; **E** C_0_/Dose of POR*1/*1 carriers versus POR*28 carriers in Caucasian recipients at different post-transplantations time courses

A significantly higher C_0_/Dose was observed in POR*1/*1 carriers compared to POR*28 carriers for recipients at 7 days post-transplantation (SMD = 0.34, 95% CI: 0.02 to 0.65, *I*^*2*^ = 84.0%). However, no significant difference was observed for the other time courses of post-transplantation (Fig. 3A). In the subgroup analysis stratified by CYP3A5 genotype, for CYP3A5 expressers (CYP3A5*1 carriers), C_0_/Dose of POR*1/*1 carriers was 22.64 (WMD = 22.64, 95% CI: 2.54 to 42.74, *I*^*2*^ = 47.2%) or 19.41 (ng/ml)/(mg/kg/day) (WMD = 19.41, 95% CI: 9.58 to 29.24, *I*^*2*^ = 73.5%) higher compared to POR*28 carriers for recipients at 3 days or 7 days post-transplantation (Fig. 3B). However, for CYP3A5 non-expressers (CYP3A5*3/*3 carriers), no significant difference was observed between POR*1/*1 and POR*28 carriers at any time course of post-transplantations (Fig. 3C). Furthermore, in the subgroup analysis stratified by ethnicity, POR*1/*1 carriers were associated with a significantly higher C_0_/Dose compared to POR*28 carriers in Asian recipients at 6 months post-transplantation (SMD = 0.96, 95% CI: 0.50 to 1.43) (Fig. 3D); however, no significant difference was observed between POR*1/*1 and POR*28 carriers in Caucasian recipients at any time course of post-transplantations (Fig. 3E).

## Discussion

The clinical use of tacrolimus is complicated by its highly variable pharmacokinetic profile, posing challenges for appropriate dosing in transplant recipients [38]. CYP enzymes, involved in the biotransformation of numerous endogenous and exogenous compounds such as steroids, fatty acids, and carcinogens [39], predominantly metabolize tacrolimus in the liver and intestine, with the CYP3A subfamily (including CYP3A4 and CYP3A5) playing a major role [40]. The proper functioning of CYP enzymes relies on the unique electron donor, POR, which affects the activities of a broad range of CYPs [41]. Thus, genetic polymorphisms affecting the expression or function of CYPs and POR may underlie the interindividual variability in tacrolimus pharmacokinetics. Several meta-analyses have investigated the association between genetic polymorphisms, such as CYP3A5*1, CYP3A4*1B or *1G, or ABCB1 3435C > T, and tacrolimus dose requirements, highlighting their significant impact on the drug's pharmacokinetics [8–11].

To date, several genetic polymorphisms including CYP3A4*22 and POR*28 had been proposed as potential predictors of tacrolimus's pharmacokinetics, but with conflicting results. As previously described, a meta-analysis demonstrated that carriers of the POR*28 variant required a higher dose of tacrolimus to achieve target levels compared to individuals with POR*1/*1. However, this meta-analysis had certain limitations, as outlined in the introduction [14]. After an extensive search of databases and careful screening of studies, six additional eligible studies [20–22, 24, 25, 37] consisting of Asians and Caucasians throughout the entire process of the transplantation were identified, ranging from 3 days post-transplantation to 1 year post-transplantation. We believed the inclusion of the supplementary eligible studies might lead to a more comprehensive result to address the limitations of the previous meta-analysis. Therefore, we conducted a meta-analysis concerning CYP3A4*22 and POR*28 polymorphism to provide an evidence-based foundation for dose adjustments based on preemptive genotyping. Our results demonstrated that weight-adjusted daily dose of CYP3A4*1/*1 carriers was higher than CYP3A4*22 carriers, especially for recipients at 1 month, 3 months, 6 months, or 12 months post-transplantations. Additionally, C_0_/Dose of CYP3A4*1/*1 carriers was lower than CYP3A4*22 carriers, especially for recipients at 3 days, 1 month, 3 months, 6 months, or 12 months post-transplantations. We also found C_0_/Dose of POR*1/*1 carriers was higher than POR*28 carriers, especially for recipients at 7 days post-transplantations. Numerous studies conducted in adults have documented a reduction in the necessary dosage of tacrolimus to achieve comparable trough concentrations as time progresses post-transplant [42–45]. The decline in tacrolimus clearance over time is typically attributed as the primary factor, although heightened bioavailability should also be taken into consideration [46]. Based on the aforementioned observations, our hypothesis posits two primary considerations. Firstly, we propose that the diminishing clearance and escalating bioavailability of tacrolimus over time post-transplant may mitigate the impact of POR genetic polymorphism on tacrolimus pharmacokinetics. Secondly, given that POR functions as the electron donor and does not exert a direct effect on CYP enzymes, its influence on tacrolimus pharmacokinetics might be comparatively weaker than that of CYP genetic polymorphism. Consequently, we postulate that the combined effect of these factors may contribute to the observed pharmacokinetic impact of POR only within the initial 7 days post-transplantation, without a sustained influence over the long-term post-transplantation period.

The impact of CYP3A5 genetic polymorphisms on the pharmacokinetics of tacrolimus has been well-documented, with CYP3A5 expressers requiring a higher dose of tacrolimus compared to CYP3A5 non-expressers to achieve similar blood concentrations [47]. To further elucidate the effect of POR*28 variant, a subgroup meta-analysis was performed based on CYP3A5 genotype, revealing that POR*1/*1 carriers exhibited higher C0/Dose than POR*28 carriers in CYP3A5 expressers, particularly for recipients at 3 or 7 days post-transplantations, while no difference was observed in CYP3A5 non-expressers, which was substantially consistent with the previous meta-analysis [14], but providing more information at other stages of transplantation. Moreover, we demonstrated the ethnicity played a role in the pharmacokinetics of tacrolimus regarding POR polymorphisms, which could be a supplementary result for the previous meta-analysis [14], as Asian recipients showed similar results to CYP3A5 expressers, particularly for recipients at 6 months post-transplantations, but no difference was observed in Caucasians with a significant publication bias for recipients at 6 months post-transplantations. Publication bias, as one of the greatest threats to the validity of meta-analysis, may result in false impressions about the magnitude and existence of an effect [48]. Therefore, the result of the subgroup analysis of Caucasians at 6 months post-transplantations between POR*1/*1 and POR*28 carriers should be interpreted with caution and warrant further validation with additional high-quality studies in the future.

The distribution of genetic polymorphisms has been reported to be significantly associated with racial diversity. For example, a remarkably different distribution of the polymorphic alleles for IL-2 genotypes was found between Black and both Asian and White populations [49]. Regarding CYP, Table 1 highlights the ethnicity characteristics in studies of CYP3A4*22 variant, which exclusively consisted of Caucasians. Consistency with the context, CYP3A4*22 variant was first reported by an allelic expression imbalance approach, explaining 12% of CYP3A4 enzyme activity variability, and was predominantly observed in Europeans and admixed Americans [50]. The minor allele frequency (MAF) of Europeans, Americans, Africans, and Asians was 5%, 2.6%, < 0.1%, and < 0.6%, respectively [51], which indicated that the variant was mainly distributed among Caucasians. Therefore, it can be further inferred that CYP3A4*22 carriers had a lower weight-adjusted daily dose and higher C_0_/Dose than CYP3A4*1/*1 carriers in Caucasians.

Additionally, the concurrent administration of additional immunosuppressive agents is imperative in the therapeutic regimen for recipients of renal transplants. Over the past decade, triple therapy regimens are widely utilized, encompassing a calcineurin inhibitor, an antimetabolite, and steroids, for both induction and maintenance purposes [52]. In our investigations, the trials incorporated predominantly adhere to a combination of immunosuppressive medications, namely tacrolimus, mycophenolate mofetil (MMF), and steroids. Steroids have served as a fundamental component of immunosuppressive therapy in organ transplantation for an extensive duration and continue to be employed for essential immunosuppression. However, the administration of high-dose steroid therapy has emerged as a significant contributor to morbidity and mortality in transplant recipients [53]. Consequently, efforts have been directed towards sparing steroids to mitigate associated co-morbidities, as highlighted in a comprehensive review [52]. In general, the current best practice for initial maintenance prophylaxis involves the use of either cyclosporine or tacrolimus-based therapy, pending the publication of long-term results utilizing newer agents [54]. Furthermore, substantial evidence exists to support the notion that MMF reduces the incidence of biopsy-proven acute rejection following transplantation, as demonstrated in large, multi-center, randomized, prospective, controlled studies [55–58]. Consequently, MMF is now commonly implemented as a primary- or second-line therapy, replacing azathioprine in the clinical practice, where azathioprine is typically reserved only for patients unable to tolerate MMF [54].

Prior research had established that acute rejection (AR) is a primary risk factor for chronic rejection and graft loss in long-term renal allograft survival [59, 60]. A meta-analysis also revealed a notable non-linear relationship between AR and tacrolimus blood concentration, emphasizing the need to maintain levels between 5–9.5 ng/ml to prevent AR [61]. Therefore, it is imperative to focus on defining the optimal initial dose and maintaining the appropriate blood concentration to prevent AR in the immunosuppressant treatment of renal transplant recipients. To achieve the desired target tacrolimus blood concentration, carriers of CYP3A4*1/*1 or POR*28 required a significantly higher dose of tacrolimus compared to carriers of CYP3A4*22 or POR*1/*1. This suggested that not only extensive metabolizers might be at higher risk of early tacrolimus underexposure leading to AR, but also poor metabolizers might be more susceptible to serious tacrolimus adverse events, which made a challenge over the rational administration of tacrolimus and long-term survival for the renal transplant recipients. Facing this challenge, upon our constant effort, it may achieve a promising settlement that determines the optimal initial dose and dose adjustment of tacrolimus based on the preemptive genotyping result combined with other individual characteristics.

### Limitations

Despite the strengths of our meta-analysis, several potential limitations should be acknowledged. First, some of the included studies reported outcomes as median and range or quartile [21, 23–25, 27, 36], which could not be directly pooled in the meta-analysis due to non-normal distribution. To address this issue, we applied a special mathematical method to estimate the mean and standard deviation [19]. While this estimating method may not represent the original data completely, it had been demonstrated to be reasonable and effective and utilized in other published meta-analyses [14, 62, 63]. Therefore, we believe that it introduced acceptable bias. Second, the number of studies investigating these genetic polymorphisms was limited, and some studies did not provide detailed information about the combination immunosuppressive therapy [22, 24]. While we included all eligible studies in our meta-analysis, this may have introduced unexpected bias and heterogeneity. However, we performed a subgroup meta-analysis excluding studies with unclear combination therapy and found consistent results with the primary analysis (Supplementary Figures S1 and S2). Thirdly, due to the nature of observational studies, there was some difference in the demography characteristics such as the mean age, percent of male, or weight between different genotypes. While random controlled trials (RCTs) may better balance confounding factors, it was not feasible to randomly group individuals based on their genotypes without interventions. Accordingly, we expected this meta-analysis to be a reasonable and reliable attempt to interpret the relationship between the investigated genetic polymorphisms and the pharmacokinetics of tacrolimus.

## Conclusions

For renal transplant recipients, CYP3A4*1/*1 carriers had a higher weight-adjusted daily dose and lower C_0_/Dose than CYP3A4*22 carriers in Caucasians. Additionally, POR*1/*1 carriers had a higher C_0_/Dose than POR*28 carriers in CYP3A5 expressers. Generally, CYP3A4*22 variant restrains the metabolism of tacrolimus, POR*28 variant enhances the metabolism of tacrolimus, and their effect should be taken into account for personalized dosing of tacrolimus in immunosuppressive therapy for renal transplant recipients. Given some limitations, further well-designed prospective studies are necessary to substantiate these conclusions.

### Supplementary Information

**Additional file 1: Table S1.** Electronic database search strategy. **Table S2.** Quality assessment of the included studies. **Table S3.** Results of publication bias assessment using the Egger's test. **Supplementary Figure S1.** Forest plots of tacrolimus’s C0/Dose of CYP3A4*1/*1 carriers versus CYP3A4*22 carriers excluding unclear combination therapy. **Supplementary Figure S2.** Forest plots of tacrolimus’s C0/Dose of POR*1/*1 carriers versus POR*28 carriers excluding unclear combination therapy.

## Data Availability

All data generated or analyzed during this study are included in this published article and its supplementary information files.
