# Supplementary material for: Effects of CYP3A4*22 and POR*28 variations on the pharmacokinetics of tacrolimus in renal transplant recipients: a meta-analysis of 18 observational studies
Source: BMC Nephrol. 2024 Feb 6;25:48. doi: 10.1186/s12882-024-03467-4 (PMC10848431; doi:10.1186/s12882-024-03467-4)
Supplement: Supplementary file 1 — Additional file 1: Table S1. Electronic database search strategy. Table S2. Quality assessment of the included studies. Table S3. Results of publication bias assessment using the Egger's test. Supplementary Figure S1. Forest plots of tacrolimus’s C0/Dose of CYP3A4*1/*1 carriers versus CYP3A4*22 carriers excluding unclear combination therapy. Supplementary Figure S2. Forest plots of tacrolimus’s C0/Dose of POR*1/*1 carriers versus POR*28 carriers excluding unclear combination therapy. [file 12882_2024_3467_MOESM1_ESM.docx]

**Supplementary files**

**Title: Effects of CYP3A4*22 and POR*28 variations on the pharmacokinetics of tacrolimus in renal transplant recipients: a meta-analysis of 18 observational studies**

Table S1. Electronic database search strategy

| SCI | |
| --- | --- |
| #1 | ((TS=(cytochrome P-450 3A4 OR cytochrome P-450 3A5 OR CYP3A4 OR CYP3A5 OR POR OR rs1057868 OR A503V)) OR TI=(cytochrome P-450 3A4 OR cytochrome P-450 3A5 OR CYP3A4 OR CYP3A5 OR POR OR rs1057868 OR A503V)) OR AB=(cytochrome P-450 3A4 OR cytochrome P-450 3A5 OR CYP3A4 OR CYP3A5 OR POR OR rs1057868 OR A503V) |
| #2 | ((TI=(tacrolimus OR prograf OR FK506)) OR TS=(tacrolimus OR prograf OR FK506)) OR AB=(tacrolimus OR prograf OR FK506) |
| #3 | ((TS=(kidney transplantation OR renal transplant OR kidney graft)) OR TI=(kidney transplantation OR renal transplant OR kidney graft)) OR AB=(kidney transplantation OR renal transplant OR kidney graft) |
| #4 | #1 AND #2 AND #3 |

| Embase | |
| --- | --- |
| #1 | 'cytochrome p-450 3a4':ti,ab,kw OR 'cytochrome p-450 3a5':ti,ab,kw OR 'cyp3a4':ti,ab,kw OR 'cyp3a5':ti,ab,kw OR 'POR':ti,ab,kw OR 'rs1057868':ti,ab,kw OR 'A503V':ti,ab,kw |
| #2 | tacrolimus':ti,ab OR 'prograf':ti,ab OR 'fk506':ti,ab |
| #3 | kidney transplantation':ti,ab OR 'renal transplant':ti,ab OR 'kidney graft':ti,ab |
| #4 | #1 AND #2 AND #3 |

| MEDLINE | |
| --- | --- |
| #1 | POR[Title/Abstract] OR rs1057868[Title/Abstract] OR A503V[Title/Abstract] OR cytochrome P-450 3A4[Title/Abstract] OR cytochrome P-450 3A5[Title/Abstract] OR CYP3A4[Title/Abstract] OR CYP3A5[Title/Abstract] |
| #2 | tacrolimus[Title/Abstract] OR prograf[Title/Abstract] OR FK506[Title/Abstract] |
| #3 | kidney transplantation[Title/Abstract] OR renal transplant[Title/Abstract] OR kidney graft[Title/Abstract] |
| #4 | #1 AND #2 AND #3 |

| Cochrane | |
| --- | --- |
| #1 | cytochrome P-450 3A4 OR cytochrome P-450 3A5 OR CYP3A4 OR CYP3A5 OR POR OR rs1057868 OR A503V in Title Abstract Keyword |
| #2 | tacrolimus OR prograf OR FK506 in Title Abstract Keyword |
| #3 | kidney transplantation OR renal transplant OR kidney graft |
| #4 | #1 AND #2 AND #3 |

Table S2. Quality assessment of the included studies

| Author, year | Clear statement of background, objectives and hypothesis | Describe the studies information | Clear eligibility criteria | Clear definition of variables | Credible method of concentration measured | Credible genetic testing method | Replicability of statistical methods | Assessment of H-W equilibrium | Sufficient descriptive demographic data | Report the withdrew person and reasons | Statement of outcome data | Funding |
| --- | --- | --- | --- | --- | --- | --- | --- | --- | --- | --- | --- | --- |
| Kuypers D,  2014 | **+** | **+** | **+** | **+** | **+** | **+** | **+** | **+** | **+** | **-** | **+** | **-** |
| Cheng F,  2021 | **+** | **+** | **+** | **+** | **+** | **+** | **+** | **+** | **+** | **-** | **+** | **+** |
| Bruckmueller H, 2015 | **+** | **±** | **-** | **+** | **+** | **+** | **+** | **+** | **±** | **-** | **+** | **-** |
| Tavira B,  2013 | **+** | **+** | **±** | **+** | **+** | **+** | **+** | **+** | **±** | **-** | **+** | **+** |
| Madsen MJ,  2017 | **+** | **±** | **-** | **+** | **+** | **+** | **+** | **+** | **+** | **+** | **+** | **+** |
| Lunde I,  2014 | **+** | **±** | **±** | **+** | **+** | **+** | **+** | **-** | **+** | **+** | **+** | **-** |
| Liu S,  2016 | **+** | **+** | **+** | **+** | **+** | **+** | **+** | **+** | **±** | **-** | **+** | **+** |
| Kurzawski M, 2014 | **+** | **+** | **-** | **+** | **+** | **+** | **+** | **+** | **+** | **-** | **+** | **-** |
| Kurzawski M, 2014 | **+** | **+** | **-** | **+** | **+** | **+** | **+** | **+** | **+** | **-** | **+** | **-** |
| Elens L,  2011 | **+** | **±** | **+** | **+** | **+** | **+** | **+** | **+** | **+** | **-** | **+** | **+** |
| Vanhove T,  2017 | **+** | **+** | **+** | **+** | **+** | **+** | **+** | **-** | **+** | **+** | **+** | **-** |
| Jonge H,  2014 | **+** | **±** | **+** | **+** | **+** | **+** | **+** | **+** | **+** | **-** | **+** | **+** |
| Elens L,  2011 | **+** | **±** | **+** | **+** | **+** | **+** | **+** | **+** | **+** | **-** | **+** | **-** |
| Zhang JJ,  2015 | **+** | **±** | **±** | **+** | **+** | **+** | **+** | **-** | **+** | **-** | **+** | **+** |
| Li CJ,  2014 | **+** | **+** | **±** | **+** | **+** | **+** | **+** | **+** | **+** | **-** | **+** | **-** |
| Phupradit A, 2018 | **+** | **+** | **±** | **+** | **+** | **+** | **+** | **+** | **+** | **-** | **+** | **+** |
| Elens L,  2014 | **+** | **±** | **+** | **+** | **+** | **+** | **+** | **+** | **+** | **-** | **+** | **+** |
| Si SH,  2018 | **+** | **±** | **+** | **+** | **+** | **+** | **+** | **+** | **+** | **+** | **+** | **+** |

“+”: detailed description; “±”: incomplete description; “-”: no description.

Table S3. Results of publication bias assessment using the Egger's test

| Comparison | time courses  of post-transplantation | VERSUS | CYP3A4*1/*1 CYP3A4*22 | POR*1/*1  POR*28 | CYP3A5 expressers | CYP3A5 non-expressers | Asian | Caucasian |
| --- | --- | --- | --- | --- | --- | --- | --- | --- |
|  |  |  |  |  | POR*1/*1  POR*28 | POR*1/*1  POR*28 | POR*1/*1  POR*28 | POR*1/*1  POR*28 |
| Weight-adjusted  daily dose | NR |  | NA | NA | NA | NA | NA | NA |
|  | 3 d |  | NA |  |  |  |  |  |
|  | 7 d |  | 0.203 |  |  |  |  |  |
|  | 1 mon |  | 0.345 |  |  |  |  |  |
|  | 3 mon |  | 0.708 |  |  |  |  |  |
|  | 6 mon |  | 0.789 |  |  |  |  |  |
|  | 12 mon |  | 0.794 |  |  |  |  |  |
| C_0_/Dose | NR |  | 0.743 | 0.229 | NA | NA | NA | NA |
|  | 3 d |  | 0.525 | 0.491 | NA | NA | NA | NA |
|  | 7 d |  | 0.509 | 0.409 | 0.953 | 0.761 | 0.520 | NA |
|  | 1 mon |  | 0.819 | 0.636 | NA | NA | NA | 0.104 |
|  | 3 mon |  | 0.145 | 0.363 | NA | NA | NA | 0.468 |
|  | 6 mon |  | 0.484 | 0.211 | NA | NA | NA | 0.037 |
|  | 12 mon |  | 0.085 | 0.445 | NA | NA | NA | 0.287 |

C_0_/Dose: dose-adjusted trough concentration; NA: not available; NR: not reported; d: days; mon: months.


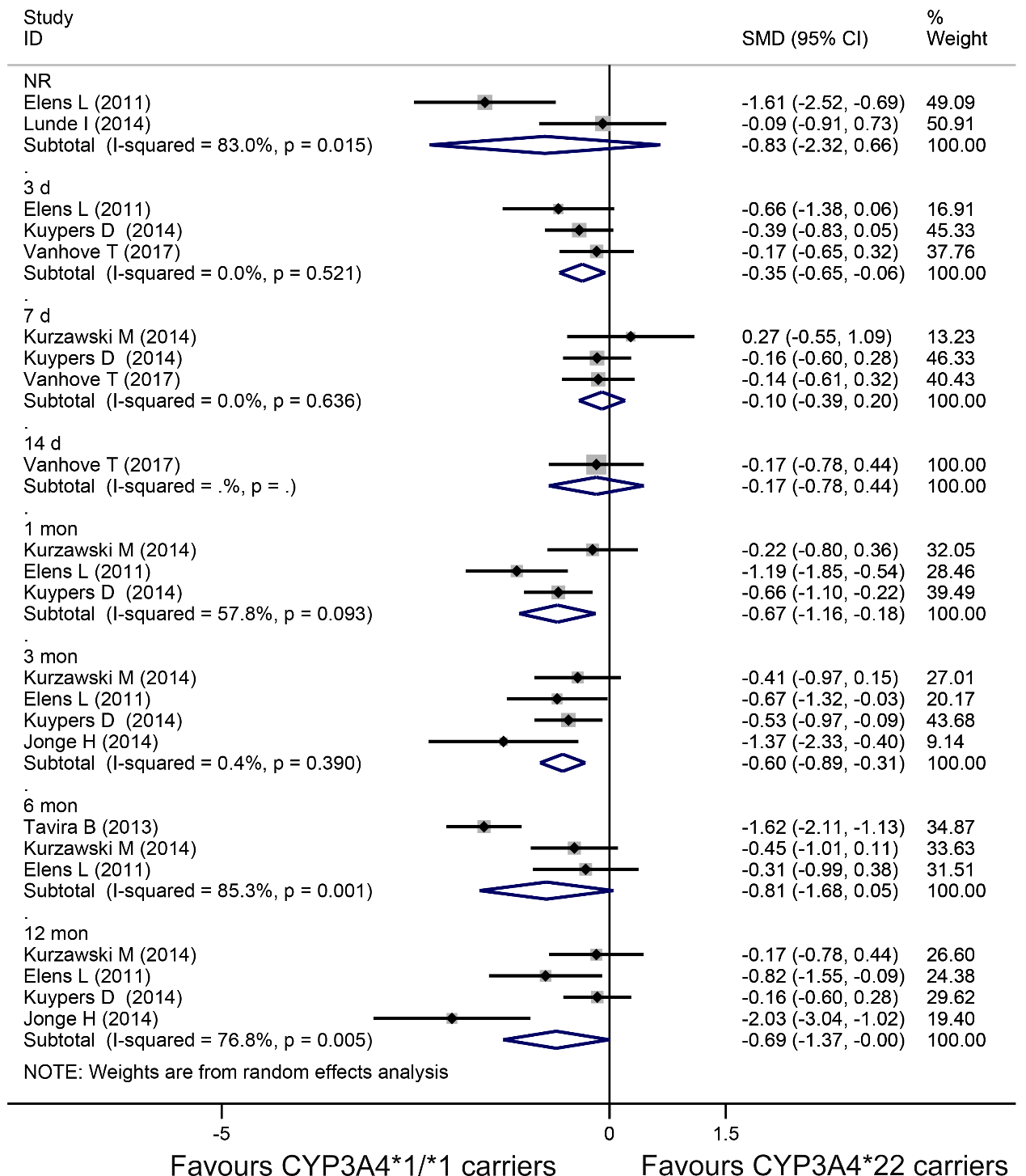


Supplementary figure S1. Forest plots of tacrolimus’s C_0_/Dose of CYP3A4*1/*1 carriers versus CYP3A4*22 carriers excluding unclear combination therapy.

C_0_/Dose: dose-adjusted trough concentration; NR: not reported; SMD: standard mean difference; CI: confidence interval; d: days; mon: months.


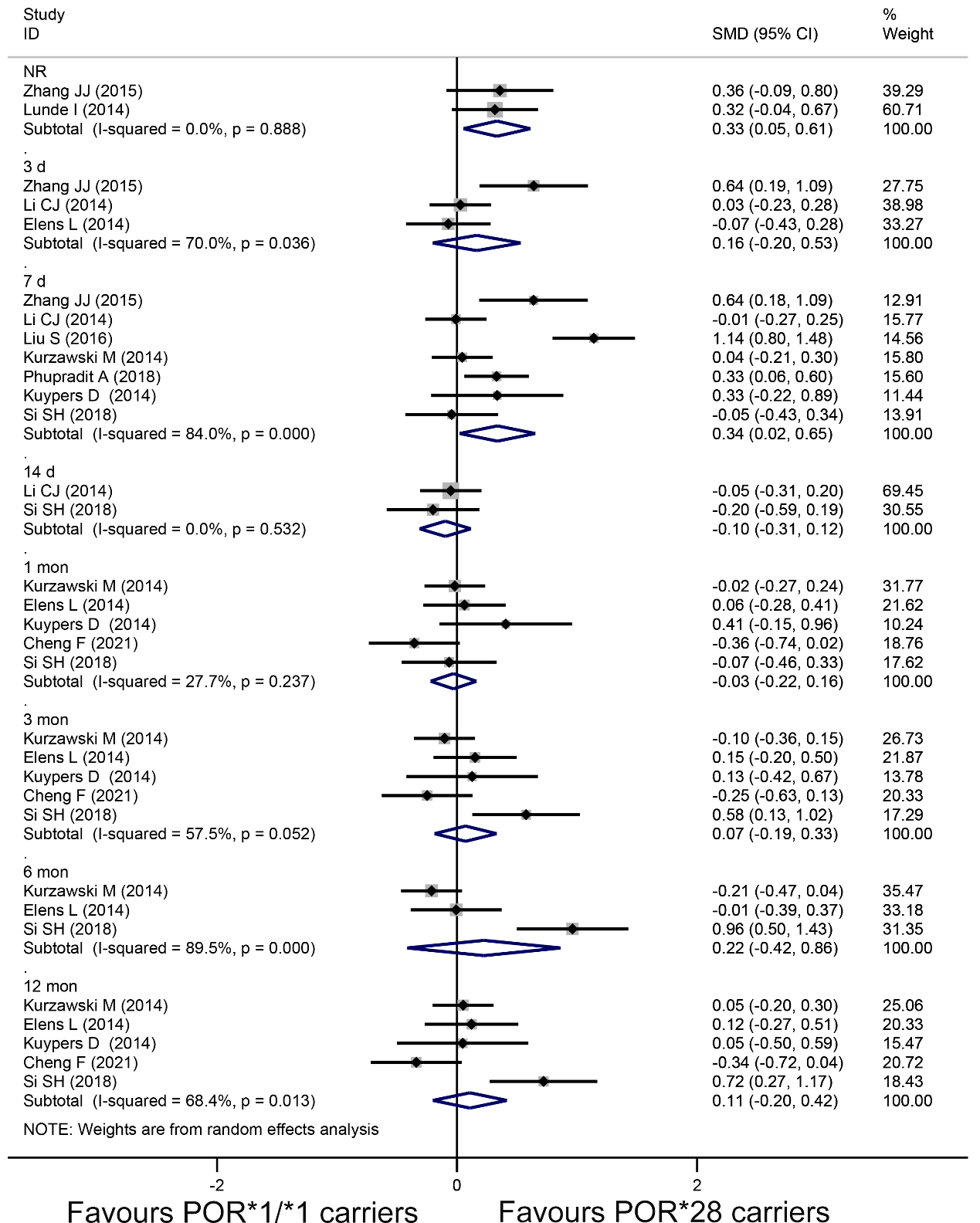


Supplementary figure S2. Forest plots of tacrolimus’s C_0_/Dose of POR*1/*1 carriers versus POR*28 carriers excluding unclear combination therapy.

C_0_/Dose: dose-adjusted trough concentration; NR: not reported; SMD: standard mean difference; CI: confidence interval; d: days; mon: months.
